# Caring for Persons Living With Dementia During the COVID-19 Pandemic: Advocacy Perspectives From India

**DOI:** 10.3389/fpsyt.2020.603231

**Published:** 2020-10-27

**Authors:** Migita D'Cruz, Debanjan Banerjee

**Affiliations:** Department of Psychiatry, National Institute of Mental Health and Neurosciences, Bengaluru, India

**Keywords:** COVID-19, coronavirus, dementia, neurocognitive, caregivers, advocacy, India

## Abstract

The Coronavirus disease 2019 (COVID-19) pandemic has presented an unprecedented threat to global public and psychosocial health. Certain vulnerable populations, especially the older adults, are at disproportionate risks both to the physiological and social effects of the outbreak. A special section among them who face unique challenges during this pandemic, are those living with neurocognitive disorders, like dementia. Limited research in the field shows ApoE4 allele to confer an increased risk for COVID-19 severity, while the behavioral problems associated with dementia reduces compliance to precautionary measures, thereby exposing them to the virus and increasing caregiver strain. Reduced healthcare access, limited resources and fear of the infection act as major barriers to dementia care during such a crisis. Besides, there are the additional burden of stigma, abuse, ageism and financial impoverishment. Institutionalization, loneliness and lack of stimulation can potentially accelerate the cognitive decline and worsen the behavioral and psychological problems. India has been one of the worst hit countries by COVID-19 and shares a significant dementia load. As the country is aging fast along with the world, this commentary reviews the risks of people living with dementia during the pandemic and discusses certain advocacies for their care.

## COVID-19: The Problem Statement

The world has endured 8 months of COVID-19 –initially reported as an outbreak at Wuhan, China in December, 2019 (1). The World Health Organization (WHO) recognized the infection as a pandemic on 11 March, 2020 (2). The first case of COVID-19 infection in India was reported on 30 January, 2020, the provisions of the Epidemic Diseases Act, 1897 were invoked on 11 March, 2020 and a national disaster announced on 14 March, 2020 (3, 4). During the pandemic, as noted by the WHO, though people of all age groups are at risk of contracting COVID-19 infection, older adults (aged 65 years and above) face a significantly higher risk of developing severe illness if they contract the disease due to the physiological changes that come with aging and other potential underlying health conditions (5). These include, but are not limited to heart disease, lung disease, diabetes mellitus, obesity, liver disease, and immunocompromised states. There is not, as of the present, any clear evidence of the interaction between the dementias and COVID-19 in older adults. However, there appears to be emerging evidence that homozygosity for the *ApoE* e4 genotype increases the risk of infection and of severe COVID-19 disease from the UK Biobank (6). Among hospitalized older adults with the COVID-19 infection (indicative of moderate to severe disease), dementias appear to be a common comorbidity and are associated with increased risk of mortality (7). The increased all-cause mortality of patients in nursing homes (among whom older adults living with dementia are over-represented) amidst the COVID-19 pandemic, reported from the United States of America (USA), United Kingdom (UK) and South Korea may also be indicative of the increased risk associated with dementia (8–10). In addition, the care as usual of older adults living with dementia is affected in several ways due to the disruption of health services and diversion of resources to contain the pandemic. This has led to barriers in the pathway to care, potentially increased time to diagnosis and management, a shortage of essential medicines and interruption of rehabilitative services. The social network has also been impacted by the physical distancing directives. The United Nations (UN) and WHO have not explicitly addressed the risk to the health of persons living with dementia. The Center for Disease Control and Prevention (CDC) has provided comprehensive guidance for the caregivers of persons living with dementia in the community as well as for the tiered management of inmates of nursing homes by health care professionals (11). In India, the Government of India and the National Institute of Mental Health and Neuro Sciences (NIMHANS), Bengaluru have issued an advisory on caring for older adults and noted in the mental health guidelines that pre-existing cognitive impairment poses unique challenges during the pandemic (12). However, there is a deficit of official information and formal guidance about how to care for persons living with dementia both from the WHO and in the Indian context. Guidance for health care professionals, persons living with dementia and their caregivers are also available from several professional bodies, advocacy and not-for-profit organizations including the International Psychogeriatric Association, Dementia Alliance International, STRiDE dementia and Dementia Australia (13, 14). Advocacy groups for Alzheimer's dementia (the most commonly identified cause of dementia) such as Alzheimer's Association(AA), Alzheimer's disease International (ADI), Alzheimer Europe and Alzheimer's Society, UK provide similar information (15). However, there is a dearth of specific and tailored guidance for other neurodegenerative disorders, with Alzheimer's disease dominating the discourse. In the Indian context, non-governmental organizations (NGOs) such as Dementia Care Notes and the White Swan Foundation provide recommendations for caregivers of persons living with dementia, though guidelines for persons living with dementia themselves are sparse—indicating a perceived lack of autonomy and agency in those living with dementia (16, 17). Further, professional bodies are yet to release detailed guidance for health care professionals involved in the care of persons living with dementia. Older adults living with dementia, their caregivers and health care professionals involved in their management in the Indian context face specific concerns during the COVID-19 pandemic that merit discussion and targeted interventions (18). The authors therefore provide below a review of these concerns, highlighting their potential challenges and advocating recommendations for care.

## Concerns

### Information, Understanding, and Comprehension

Persons living with dementia bear cognitive deficits with regard to their working memory, encoding, information processing, comprehension, recall, language, reasoning, planning, and judgement. These deficits worsen with the severity of dementia and the relative degree of deficits vary with the nature of the dementia (19, 20). The nature of these deficits may impair persons living with dementia from understanding the nature of the COVID-19 pandemic and the guidelines placed in response to it, much less the need to comply with it. Persons living with dementia in the community will find it harder to comply by shelter in place, social distancing, usage of masks and gloves or sanitation. The difficulty in comprehension of information may worsen with the large volume of information pouring in each day related to the pandemic and the frequent changes in guidelines. Particular phenomena, such as confusional arousal, fluctuation in orientation, glycaemic disturbances, electrolyte disturbances, sun downing, visual and auditory impairment might worsen comprehension deficits. While much has been made of a possible worsening of anxiety, agitation, restlessness, aggression and other positive problematic behavioral symptoms—all persons with dementia are not alike and may react to the barrage of information about the pandemic in differing ways. Behavioral and psychological symptoms associated with dementia (BPSD) may make it difficult for older adults to comply with precautionary and therapeutic measures, though withdrawal may manifest as often as agitation. A concern unique to persons living with dementia in India is the multiplicity of languages, ethnicity and culture within a single country (17). Much of the information available is directed toward an English speaking urban audience—with only limited penetration into vernacular languages and rural or underprivileged populations. There is also limited fact-checking and verification of the authenticity of translated information, with scope for interpretation in differing ways—which can worsen ambiguity and anxiety.

### Morbidity and Mortality Due to COVID-19 Infection

Emerging data from the UK Biobank Cohort reveals that persons living with dementia are over-represented among older adults with symptomatic or severe SARS-CoV-2 infection, in those requiring hospitalization, mechanical ventilatory support and those who have succumbed to the infection (6). Older adults with dementia are often frail, with impaired mobility, respiratory reflexes and regulation, immunocompromised, and likely to have multi-morbidity and poly-pharmacy—all of which are adverse prognostic factors for any infection.

Based on data from the Chinese Centre for Disease Control and Prevention, the age-adjusted fatality rate in 60–69 years is 3.6% which rises to 18% above 80 years (21). Further, age is also an independent risk-factor for non-pulmonary involvement and septicaemia, that can add to the morbidity. An age-wise comparative study by Liu et al. (22), reported three times increased mortality risk in people above 55 years who are affected by the outbreak. Ioannidis et al. (23) in their cross-sectional survey of 14 countries showed that people lesser than 65 years of age have 10-fold lesser risk of morbidity and mortality in India. The authors also highlighted the lack of systematic age-specific data in the developing countries. Further, more than 60% of the dementia cases are from low and middle-income countries (LMIC). This makes the “dual” burden of “age” and “cognitive impairment” all the more relevant in a populated and diverse LMIC country like India.

Further, there is also emerging data from the UK Biobank cohort, again, that the *ApoE* e4 genotype, associated with both delirium and dementia, particularly a 14-fold high risk of Alzheimer's disease confers a higher risk of infection. It appears to increase risks of severe COVID-19 infection independent of pre-existing dementia, cardiovascular disease, and type-2 diabetes. The *ApoE* e4 gene is co-expressed with ACE2 receptor in the respiratory epithelium and in type II alveolar cells as well as neurons and glia, which may indicate a possible inflammatory pathogenesis (6). The *ApoE* e4 genotype is less frequent in the Indian population than in people of European ancestry, though there is increased frequency of occurrence in persons with Alzheimer's dementia and vascular dementia (24). Hypoxia associated with COVID-19 infection can induce delirium—a poor prognostic factor for the infection, pre-existing dementia and overall all-cause mortality. India is facing a potential shortage of ventilators and intensive care as cases are on the rise—a shortage that will hit persons living with dementia harder (25). There has been an increase in mortality in nursing homes from USA, UK and East Asian—both due to the rapid spread of COVID-19 infection within a closed population and sudden, unexplained mortality—which may be partly indicative of the increased biological vulnerability of persons living with dementia (9). This is of relevance to and concerning in India, where there is a paucity of data from nursing homes. Palliative care in dementia can be considered to be a second priority at times of pandemic crisis.

### Potential Worsening of Dementia With COVID-19 Infection

A possible increase in new onset ischemic stroke, intra-cranial hemorrhage and worsening of pre-existing cerebrovascular disease, including vascular dementia due to the inflammatory cascade triggered by the cytokine storm during NeuroCovid Stage II and III has been postulated by neurologists (26, 27). It has also been postulated that the inflammatory cascade may worsen other neuro-inflammatory and degenerative disorders such as Parkinson's disease and multiple sclerosis. This hypothesis is partly based upon the epidemic of encephalitis lethargica after the 1918 influenza pandemic. Similar observations of histological and motor symptoms of Parkinson's disease have been noted after H1N1 epidemic and outbreaks of West Nile Virus, Japanese Encephalitis B, Coxsackie Virus and HIV (28).

Cytokine imbalance is one of the many factors involved. Delirium has been reported as one of the commonest neuropsychiatric manifestation of COVID-19, pre-existing cognitive deficits being one of the important and obvious risks. Urinary retention, medical comorbidities, polypharmacy, cytochrome interactions, insomnia, tissue hypoxia, desaturation and use of hydroxychloroquine have been proposed as the risk factors for confusional states and worsening cognitive status in COVID-19 infections (29). In both animal and human models, the possible neural spread of SARS-CoV-2 has been linked to Angiotensin Converting Enzyme (ACE)-2 binding in respiratory and olfactory epithelium, invasion of the pyriform cortex and dissemination in hypothalamus, thalamus, parahippocampal cortex, basal ganglia, and amygdala (30). These brain regions are also implicated in neuro-degenerative disorders like dementia and a bi-directional relationship can thus be hypothesized. In an ecological study by Azarpazhooh et al. (31), healthy life-expectancy and dementia disability adjusted life years (DALY) were significantly related to the COVID-19 caseloads and mortality. Studies have also shown significant impact of COVID-19 related lifestyle changes, social isolation, loneliness and quarantine measures on the behavioral and psychological symptoms of dementia (BPSD), especially sleep disturbances, anxiety, depression, agitation and wandering (32, 33)

### Loneliness and Social Isolation

Under-stimulation as a result of reduced social interaction may accentuate cognitive decline in those vulnerable. Lockdowns all over the world and in India limit opportunities for physical and cognitively stimulating activities. There is also a restriction on visits by friends and family in the community, in acute care and in long term care homes, including nursing homes. Social isolation and loneliness are likely to exacerbate cognitive deficits in persons living with dementia (34, 35). Services for older adults in the community and in hospitals are often segregated, which while allowing specialist care and reducing the waiting period, can worsen under-stimulation. Digitization has been postulated as an alternative to interaction and stimulation. However, India, where functional digital illiteracy is estimated to be above 90% is likely to have poor penetration of digital services in persons living with dementia—unless aided by a formal or informal caregiver (36). Poverty, lower education levels, unemployment and rural living make underprivileged populations less likely to have digital penetrance. Further, there are lesser digital services available in the vernacular language and which are respectful of diversity in culture, religion and ethnicity with aging (gero-diversity) (37).

### Delay and Barriers in Pathways to Care for Dementia

The cessation of non-essential health care services, including dementia care and rehabilitation, and the diversion of health care resources toward pandemic control is a potential source of delay in and barrier to diagnosis, treatment and care of persons living with dementia in India. Older adults are encouraged to stay at home, and delay non-emergency consultations—with an increase in time to care for new onset dementias and barrier in the continuity of care of patients already on treatment (25). Structural procedures such as travel restrictions also limit the ability of patients with dementia to travel to hospitals—a factor of importance in India, where most of dementia care is available only in tertiary care facilities and in large urban areas (16). A delay of a few weeks to months may prove to be critical for persons living with dementia—especially young onset and rapidly progressive dementias, where a critical window of opportunity is lost (25). Another potential barrier to care is economic. In India, where 80% of care is delivered by the private sector, and dementia medicines and cognitive re-training do not come under subsidized state care—the care of a person living with dementia has always been an expensive affair for the average Indian household—costing between 3,000 and 15,000 Indian National Rupee (INR) a month at a conservative estimate (38). The only potential benefits available for senior citizens are old age pensions and disability benefits of 1,000–1,200 INR a month—with poor and inconsistent coverage which is inadequate to offset the costs of caring for a person with dementia. These financial constraints and loss of income to household represented by the pandemic and lockdown may further place critical care for dementia—including essential medicines out of the pocket of families (38).

### Caregiver Burnout and Strain

Most of dementia care in India is delivered in the community and by informal caregivers- usually family and most commonly women (38). Constraints placed on persons living with dementia may hasten cognitive decline and worsen BPSD, worsening burnout of caregivers. Further, work from home and school vacations mean that family members now spend more time with persons living with dementia in close contact. This has potential to be a rich and fulfilling interaction but may also be a source of strain for caregivers who find themselves performing a double shift—professional and personal (39). Caregivers of persons with dementia usually report turning to informal work, or work with more flexible times, though lower paid, in order to balance their caregiving responsibilities. In India, 97% of paid work is estimated to be in the informal sector. The International Labor Organization estimates that poverty is estimated to double as a results of the pandemic (40). The caregivers of persons with dementia are over-represented in this sector and are vulnerable to a loss of income. These economic constraints is likely to interact with the physical limitations during the pandemic to compound dementia care and caregivers. Spouses are the most common caregivers of persons living with dementia and have to deal with increased vulnerability to the infection (38). Another concern for persons living with dementia is the potential separation from their spouse during quarantine or hospitalization, a terrifying experience for the couple. Spousal caregivers may also have to address the issue of who will take over care of the person with dementia in the event of their demise (41). The restrictions on advance directives, do not resuscitate choices, interment services and funerals are also potential sources of distress for persons with dementia and their spouses.

### Abuse and Fraud

Reports of elder abuse has increased 10-fold across the world and 4-fold in India—including the NIMHANS elder helpline (42, 43). Persons with dementia are particularly vulnerable to abuse due to higher dependency needs. Further, the most common source of abuse is the caregiver, leading to the postulation that elder abuse may be a marker of caregiver burden. A HelpAge study in 2018 estimated 25% of Indian older adults had undergone elder abuse at some point, though conclusive data on persons living with dementia was lacking. This vulnerability is likely to increase (44). The limited availability of reporting and social welfare services in India—as well as the interruption of these services due to the pandemic is another risk factor. Persons living with dementia are also vulnerable to fraud, especially digital fraud, which is on the rise across the world during the pandemic (45). Again, conclusive data from India is lacking, but this is a potential area of vulnerability that would benefit from monitoring.

### Health Care Professionals Involved in the Care of Persons Living With Dementia

Health care professionals involved in the care of persons living with dementia are struggling to provide appropriate care while maintaining the safety and welfare of their patients. Often, this care is delivered in stressful and resource poor settings, with inadequate structural provisions and safety equipment (46). Further, several health care professionals specialized in dementia care have been diverted to other health services for pandemic control—particularly in the public sector and must also deliver infection control. Masks and personal protective equipment used during consultation impair easy recognition of healthcare providers by persons with dementia and limit paralinguistic and non-verbal cues—a barrier to effective communication with the person. This may be accentuated in case of auditory and visual impairment (47). Primary health care is an important alternative, however, limited training in dementia and incomplete coverage of the population prevents it from being an effective alternative to tertiary referral. The possibility for the potentially inappropriate use of medication in persons with dementia is higher during pandemic control, when the focus is symptom control rather than comprehensive management.

## Recommendations

Based upon these concerns in the care of persons living with dementia in India, the authors highlight some potential ways to address these, which may be incorporated into pandemic control. The comprehensive care of persons living with dementia, including appropriate safety and psychosocial considerations are an important component of public health. Unfortunately, this population has been left out of pandemic preparedness policies leading ambiguity in the guidelines for their care. Psychological well-being comes with physical security and the precautionary measures against the outbreak need to be well-guided and supervised by the caregivers tailored to the cognitive needs of people living with dementia. Acceptable standards of care need to be maintained considering the special needs of this population during such crisis, and the approaches that can be attempted differently are highlighted in brief:

- Information delivered to persons living with dementia must be performed slowly, with frequent pauses, in short and simple sentences with use of audio-visual aids. Communication can be attempted when the person is at their cognitive best during the day. Patients may be encouraged to revisit the information at periodic intervals. Pre-recorded audio and video material as well as simple infographic visual charts can act as cues help reinforce information.
- Psychological preparedness among the caregivers of people living with dementia is of paramount importance for the continuity of care. During such a contagious pandemic, there is always a possibility that the caregiver might himself/herself get infected with the pandemic and is unable to provide the required support. Alternative sources of care including friends, relatives, volunteers need to be planned and prepared for in advance. Tele-consultations are always a feasible option, especially as the National Institute of Mental Health and Neurosciences along with the Indian Psychiatric Society (IPS) has recently released the telepsychiatry guidelines, the first of its kind in India (48). Such guidelines set a standard of care for the physicians for virtual service delivery, which can be harnessed effectively in dementia care.
- Information to persons living with dementia is better delivered in the vernacular language, with tailored socio-cultural contexts. Information must also be relevant to the context of the individual. Meri Yaadein in the United Kingdom has provided excellent resource material on how practicing Muslims of the South East Asian ethnicity living with dementia and observing Ramadan during the pandemic may be handled in a culturally and religiously sensitive manner. Similar Indian material on COVID-19 and its impact upon the ethos and diversity of Indian life may help persons with dementia and their caregivers navigate the pandemic.
- Information, Education and Communication (IEC) for persons with dementia may be halted if the person expresses anxiety or becomes agitated and appropriate reassurance provided in a calm and soothing manner. Media has an important role to play for this awareness, but at the same time people with cognitive deficits are more prone to misinformation. Hence the authenticity of the sources need verification and guidance. Relevant and tailored information is better than an “information overload.”
- A lower index of suspicion and testing of infection for persons living with dementia, their caregivers and inmates of nursing homes may be beneficial effective care and reduce morbidity and mortality.
- Research in the Indian context would fill an existing lacuna in the field. It may go a long way toward examining the potential interactions between the COVID-19 infection and neurodegenerative disorders and may guide informed care. This is of particular relavence with over two third of persons with dementia living in LAMI countries like India with numbers that are projected to increase with time.
- Attempts should be made to decentralize dementia care and integrate it into community health care and the district mental health programme (DMHP) to address barriers and delay in care.
- An early resumption of dementia care with due precautions and integration with tele-medicine where possible may cut down delay in diagnosis and care.
- Subsidization of dementia care where feasible, with coverage of investigations, medicines and training in welfare schemes such as the National Mental Health Programme (NMHP), National Policy on Senior Citizens and Ayushman Bharath would make dementia care during the pandemic more affordable.
- Interaction with family, friends and the community in a safe manner, regular physical and mental exercise and adequate nutrition and fluid intake in persons living with dementia need to be encouraged. Social connectedness is vital and digital services can be used to the extent feasible. Meeting or interacting with their loved ones or even pets, albeit virtually, can help both cognitive issues and BPSD. Simple steps like music, group activities, prayers, spending more time with people living with dementia can increase the “contact time” and help in reducing the behavioral issues.
- Addressal of caregiver burden, psychosocial support in the community, provision of social security and remuneration of informal caregivers is recommended. Some states such as Kerala provide remuneration of around 600 rupees per month to female unpaid caregivers of persons with mental or physical illness—a model which may be of use with rising unemployment. Caregivers need to be counseled about their own “respite” time to prevent burnout. The National mental health counseling helplines need to be availed and integrated with the elder service helplines.
- Helplines, legal aid and social services to protect vulnerable persons from abuse and fraud and provide remedial aid is recommended. This can be combined with the education of caregivers and the community. These measures would help address abuse and fraud targeting persons living with dementia—who currently have inadequate safeguards under India law. Early detection of abuse, legal hassle free reporting and appropriate mental health care of the abuse victims are essential. Training for home-based management of behavioral symptoms can help prevent unwarranted abuse.
- Advance directives may be discussed with persons living with dementia and their caregivers, particularly spouses to ensure their wishes are honored in management of the infection and of dementia.
- Health care professionals can be encouraged to provide dedicated care to persons living with dementia, where feasible and with appropriate precautions. Voice modulation and non-verbal communication may be required to traverse the barrier provided by masks and face-shields. A single point contact of care with a familiar health care professional may reassure the person living with dementia considerably. Family physicians thereby play an important role here. Addressal of stress and burnout in health care professionals, including peer and supervisory support may also make care more effective. The primary care health workers may benefit from added expertise in dementia care, and tele-training can be enabled during the current times for integration of various levels of health care.
- Most importantly, it's a collective responsibility at all levels of stakeholders to identify the needs of people with cognitive impairment, their caregivers and establish planned strategies for their assistance, improvisations that can be used even post-pandemic. Few non-governmental organizations like the Alzheimer's and Related Disorders Society of India (ARDSI), often partners with Governmental initiatives for community dementia care and support. ARDSI has been providing support and guidance via telephonic, video and social media platforms (49). The Psychiatric Social Work team along with the District Mental Health Programme (DMHP) officials can ensure home-visits and medication availability for compliance.
- The above-mentioned provisions of care are only possible taking into account the respect for autonomy and dignity in people living with dementia, fostering independence, hope and empathy. The “need to care” should not be mis-perceived as “coercion” and “covert abuse.” Engaging them in conversations related to their health and safety as much possible is helpful.

## Conclusion

India is aging fast. It is estimated that 20 percent of the population will be over 65 years of age by 2050 (50). A projected 5.3 million people are affected with dementia in India at present. The Global Strategy and Action Plan on Aging and Health was adopted by the World Health Assembly in 2016 to prepare for the Decade of Healthy Aging which began in 2020 and is expected to last till 2030 (51). In lines with the same, people affected with dementia also deserve a “humanitarian” and “right-based” approach to age and live a healthy life. Neurocognitive disorders (including the dementias) are a significant co-morbidity that increases in prevalence over the lifespan. In this review, we have attempted to discuss how the persons living with dementia face dual risks due to both age and cognitive decline, which are accentuated by the pandemic. Sensory deficits, behavioral problems, caregiver strain, associated abuse and neglect, lack of recognition of autonomy, limited opportunities for advocacy and administrative apathy are important social problems that add to recognized biological risks. Ageism is a form of stigma in itself which may further compound pandemic related stigma in those affected. Bearing in mind this problem statement, the authors suggest a comprehensive care model with an integrated bio-psycho-social approach to address the needs of the persons with dementia and their caregivers in the Indian context. Health and social services can improve from continued training and increased sensitivity to the concerns of persons living with dementia. Further, India is still in the initial stages of the pandemic. Greater psychosocial morbidity is expected in the months to come. Systematic research into the experiences of persons living with dementia can help tailor effective healthcare. Caregivers and health professionals are important allies to persons living with dementia and can contribute to advocacy and care. As an ending note, the authors would like to state that pandemic control in India can be best achieved when persons living with dementia are made part of and advocates for, rather than mere recipients of care.

## Data Availability Statement

The original contributions presented in the study are included in the article/supplementary material, further inquiries can be directed to the corresponding author/s.

## Author Contributions

Both the authors were involved in conceptualization, literature review, and drafting the manuscript. The final version was read and approved by both the authors.

## Conflict of Interest

The authors declare that the research was conducted in the absence of any commercial or financial relationships that could be construed as a potential conflict of interest.
